# Variables affecting pricing of orphan drugs: the Italian case

**DOI:** 10.1186/s13023-021-02022-w

**Published:** 2021-10-19

**Authors:** Claudio Jommi, Elisabetta Listorti, Federico Villa, Simone Ghislandi, Armando Genazzani, Agnese Cangini, Francesco Trotta

**Affiliations:** 1 Cergas (Centre for Research on Health and Social Care Management), SDA Bocconi, Bocconi University, Via Sarfatti 10, 20136 Milan, Italy; 2 Aifa (Italian Medicines Agency), Via del Tritone 181, 00187 Rome, Italy; 3 Department of Social and Political Sciences, Bocconi University, Via Rontgen 1, 20136 Milan, Italy; 4 Department of Pharmaceutical Sciences, Università del Piemonte Orientale, Largo Guido Donegani, 2, 28100 Novara, Italy

**Keywords:** Orphan drugs, Rare diseases, Health technology assessment, Pricing, Italy

## Abstract

**Background and aim:**

Evidence on determinants of prices for orphan medicines is scarce and not available for Italy. The aim of this paper is to provide an evidence on variables affecting the annual treatment cost of orphan drugs in Italy, testing the hypothesis of a negative correlation with the dimension of the target population and a positive correlation with the added therapeutic value of the drug and the quality of the evidence of pivotal studies.

**Methods:**

Drugs with a European orphan designation reimbursed in Italy in the last 6 years (2014–2019) were considered. Univariate, cluster analysis and multiple regression models were used to investigate the correlation between the annual treatment cost and, as explanatory variables, the dimension of the target population, the existence of Randomized Clinical Trials as a proxy of the quality of the pivotal studies, the added therapeutic value.

**Results:**

In the univariate analysis prevalence and added therapeutic value, as expected, have a negative and positive correlation with cost respectively. The correlation with RCT is not significant. In the multivariate model, coefficients for prevalence and added value are confirmed but for the latter are not significant anymore. We also found, through an interaction analysis, that the existence of an RCT has a positive impact on annual treatment cost when the target population is very small.

**Conclusions:**

Our results suggest that value arguments and sustainability (dimension of the target population and its impact on budget impact) issues are considered for orphan drugs pricing: the role played by sustainability is systematically supported by our results. A more transparent and reproducible price negotiation process for orphan drugs is needed in Italy. This paper has contributed to highlight the implicit drivers of this process.

## Background

Market access for pharmaceuticals in Italy is regulated by the Italian Medicines Agency (AIFA) and the Regions.

Price and reimbursement (P&R) for new medicines and indications are negotiated by AIFA and the relevant company on the grounds of a dossier. This dossier is sent by the company after the publication of the European Commission Deliberation (or immediately after the positive opinion of the CHMP—Committee for Medicinal Products of Human Use of the European Medicines Agency—for orphan medicines and drugs with an exceptional therapeutic value). The dossier includes information on the target disease (target population and disease severity), the level of unmet need, the added therapeutic value, the impact on pharmaceutical budget (possibly integrated by a cost-effectiveness analysis and a health care budget impact analysis), and prices in other countries [1, 2].

Pharmaceutical companies may also apply for the innovativeness status for their product/indication, in parallel with P&R. AIFA provides for full or conditional innovativeness (or may reject the request). Full innovativeness status provides the relevant medicine/indication with some advantages from an access perspective. These advantages consist of two dedicated funds (one for cancer treatments and the other one for all other innovative medicines) and immediate patient access at the regional level. For other medicines clinicians should wait for the inclusion into the regional formulary, if any, to prescribe the drug for the target population. The latter advantage is provided also in the case of conditional innovativeness. The criteria to get innovativeness status are the unmet therapeutic need, the added therapeutic value, and the quality of the evidence [3]. To appraise the quality of the evidence, AIFA has chosen the Grading of Recommendations Assessment, Development, and Evaluation (GRADE) method [4]. Innovativeness appraisal reports are published on AIFA’s website [5]. Seventy-seven reports have been published so far: 30%/36%/34% medicines/indications were appraised innovative, potentially innovative and not innovative, respectively. Innovative status has no direct impact on P&R negotiation. And, interestingly, innovative medicines have shown a greater than average difference between the list price proposal submitted by the industry and the final negotiated price [2].

Managed Entry Agreements (MEAs), including discounts, financial-based agreements (e.g. price–volume agreements), and outcome-based agreements (e.g. performance-linked reimbursement) are extensively used in P&R negotiation [6–8]. MEAs negotiated with AIFA and possible additional discounts at the regional and local levels make the actual price charged to payers lower than the published list price, with a mean difference of 32% [9].

Patients may also benefit from two early access programs covered by the Italian National Health Service, i.e. medicines reimbursed before approval. The first one concerns medicines for which there is evidence at least from phase II studies, showing favourable clinical efficacy and safety data in settings where there are no valid therapeutic alternatives or the existing alternatives are more expensive. The second one regards orphan medicines and other medicines for rare diseases and severe diseases [10].

Once the P&R process is concluded, the actual access of the drug depends on Regions, which are accountable for the health care budget. The Regional Governments have implemented different pharmaceutical policies. These policies include binding regional formularies, cost-sharing, guidelines on drug procurement, direct distribution of drugs by hospitals, clinical governance and prescription targets, regulation of information and advice provided by pharmaceutical companies’ sales representatives [11].

In 2019, orphan medicines accounted for 5.6% of public pharmaceutical expenditure in Italy [8]. Their market share systematically increased from 2010 to 2019, similarly to what happened worldwide [12]. There are no specific policies on access for orphan medicines in Italy, apart from the above-mentioned early access program and accelerated submission of the P&R Dossier. Orphan drugs are formally subject to the same P&R process as other medicines, although different contributions have advocated for specific P&R processes and criteria. The European Working Group for Value Assessment and Funding Processes in Rare Diseases (ORPH-VAL) recommended that (1) prices for orphan medicines are determined by considering the magnitude of the product value in light of price-value precedents for other specialised technologies and medicines; (2) P&R status is modulated to reflect considerations beyond the orphan status, such as societal preferences, rarity, sustainability (budget impact) of innovation in rare diseases; (3) P&R decisions are aimed at contributing to the right balance between enabling sufficient revenue generation to stimulate new investment in research on rare diseases and attract private funding while maximizing the value for money for healthcare systems [13]. A more pragmatic paper illustrated which variables could be considered to determine the prices for orphan drugs, including the rarity and severity of the target disease, the quality of the evidence provided for marketing authorization, the level of unmet need, the impact on condition/disease modification, the manufacturing complexity, and the number of indications approved for the same drug [14].

Orphan drugs share also the same rules of other medicines to get innovativeness status; however, it has been stated that medicines for rare diseases may be recognized innovative also with a low quality of evidence [3] since they rarely fit with GRADE requirements.

Finally, regional policies are applied to orphan drugs as well as other medicines: e.g. there is no accelerated pathway for the inclusion of orphan medicines into the regional formularies.

The empirical evidence on variables influencing orphan drug prices is quite scarce. There is some evidence showing a price–volume trade-off, with higher prices for orphan versus non-orphan drugs [15] and ultra-orphan versus orphan medicines [16]. A recent paper investigated the determinants of orphan drugs prices in France through a regression model [17]. The multivariate analysis found out a significant correlation between the annual treatment cost of orphan drugs and: (1) the availability of alternative treatment options (higher annual treatment cost in case of no alternatives); (2) the added therapeutic value scored by the Transparency Commission (higher annual treatment costs for major and important added therapeutic value); (3) the existence of a comparator in the pivotal clinical trial. The correlation with the prevalence was found negative in the univariate analysis and positive, but not significant, in the multivariate analysis.

The aim of this paper is to investigate the determinants of orphan drugs annual treatment cost in Italy, where the empirical evidence is limited to the role played by the disease prevalence [15, 16]. Our empirical findings shed new light on which variables are actually considered when prices for orphan drugs are set.

## Methods

Medicines covered by the analysis are the ones that obtained European orphan designation (at the time of the submission of the P&R request in Italy) and have positively concluded their reimbursement procedure in Italy over the last 6 years (2014–2019). The information system in AIFA did not allow us collecting data for previous years. If the drug had more than one indication, the first one in order of approval time was considered; if two or more indications were approved simultaneously, two or more observations were used for the analysis if the annual treatment costs were different (due to different dosages per indication). If the annual treatment costs were the same, we considered the indication with the highest prevalence.

The dependent variable is the orphan medicine annual treatment cost. The annual treatment cost was calculated on the grounds of the net price (i.e. including hidden discounts and the expected impact of outcome-based managed entry agreements), dosage and treatment schedule reported in the Summary of Product Characteristics - SmPC (including loading dose), one year of treatment duration unless a shorter time is envisaged by the SmPC (e.g. for one-shot therapies). If there are different prices for reimbursed presentations (and therefore a different annual treatment cost), the highest one was chosen. To estimate the impact of financial-based and outcome-based MEAs, the Java program “Plot Digitizer” was used [18].

Three main independent variables were analysed in their impact on the cost: (1) the prevalence of the disease, as it was reported by the pharmaceutical companies in the P&R Dossier; (2) a variable detecting the quality of the pivotal studies, as they were reported on the EPAR document (European Public Assessment Report), i.e. whether they are designed as Randomised Clinical Trials (RCT); (3) the added therapeutic value (ASMR—Amélioration du Service Médical Rendu) as it was graded by the French Transparency Commission (I—Major innovation: innovative product with substantial therapeutic benefit; II—Important improvement in terms of therapeutic efficacy and/or reducing side effects; III—Moderate improvement in terms of therapeutic efficacy and/or utility; IV—Minor improvement in terms of therapeutic efficacy and/or reducing side effects; V—No improvement over existing options but still can be recommended for reimbursement [17]) and published on the Avis document [19].

We could not rely on the Italian evaluation of the added therapeutic value, since it is appraised, graded and reported only for medicines whose marketing authorization holder applies for innovativeness status [20]. The French evaluation was used since the price and reimbursement system in Italy is more similar to the French than to the German one, which is the only country with France where the added therapeutic value is published. Furthermore, in Germany the added therapeutic value is not necessarily appraised for orphan drugs [19].

Our hypothesis was of negative correlation for the prevalence-cost relation, because of the expected trade-off between the unit price and volumes, while we expected a higher added value to bring higher prices in the negotiation process. Due to its relevance in terms of quality of the scientific evidence produced, we also expected RCT pivotal studies would correspond to higher costs; however, since these characteristics are not officially included in the cost decisional process, we were dubious about its statistical significance. Other data were retrieved from pivotal clinical studies, including the phase in the clinical development process, the primary endpoint (final vs. surrogate), and other elements of the study design (single/arm vs double/arm, placebo-controlled vs head-to-head, double-blind vs. open). Given the inevitably small sample size of the database, a parsimonious model was preferred, and we decided to focus on the RCT, considering it the best proxy of the quality of the studies.

We also collected other data on orphan drugs reimbursement status as control variables, i.e. the year when the price and reimbursement decision was published, the ATC (Anatomical Therapeutic Chemical) of the indication (L—Antineoplastic and immunomodulating agents vs others), and the class of reimbursement (H that includes medicines reimbursed only in hospital settings; A that includes drugs reimbursed also in the retail market).

Other possible explanatory variables are the availability of alternative treatments (level on unmet need), their prices (that could represent a benchmark for pricing the new comers) and disease severity [17]. We have decided not to include them because their measurement is quite controversial. Alternative treatments, if any, could be drugs used off-label or drugs used for similar, but not identical, indication. Disease severity is not easy to categorise and the distinction between severe/not severe disease, made in other contributions on the grounds of the Public summary of opinion on orphan designation [17], seems too simplistic and would have add another binary variable.

Factors influencing the cost were analysed through multiple approaches. The association between each explanatory variable and the cost was firstly observed with graphs of descriptive statistics, together with t-tests and correlation tests. Subsequently, a cluster analysis was run, whose rationale was to identify groups of drugs having specific characteristics (corresponding, of course, to the independent variables of our analysis) and significantly different levels of annual treatment costs. Given that we treated ASMR as a continuous variable while the same does not hold for prevalence and RCT, we faced a clustering data of mixed types calling for the use of a proper distance metric, such as the Gower distance. Once obtained the dissimilarity matrix, we did clustering from it, using the Partition Around Medoids algorithm. To select the optimal number of clusters we relied on silhouette width approach, an internal validation metric which is an aggregated measure of how similar observation is to its own cluster compared to its closest neighbouring cluster. In a further step, we performed a series of regression analyses: first, we focused on (three) univariate regressions, where only one variable was used; then, we performed one multivariate regression with all the three variables included and another where we added also interaction terms. Eventually, we repeated the analysis with the set of the above-mentioned control variables (year of negotiation, reimbursement class, ATC class).

## Results

**Fig. 1:**
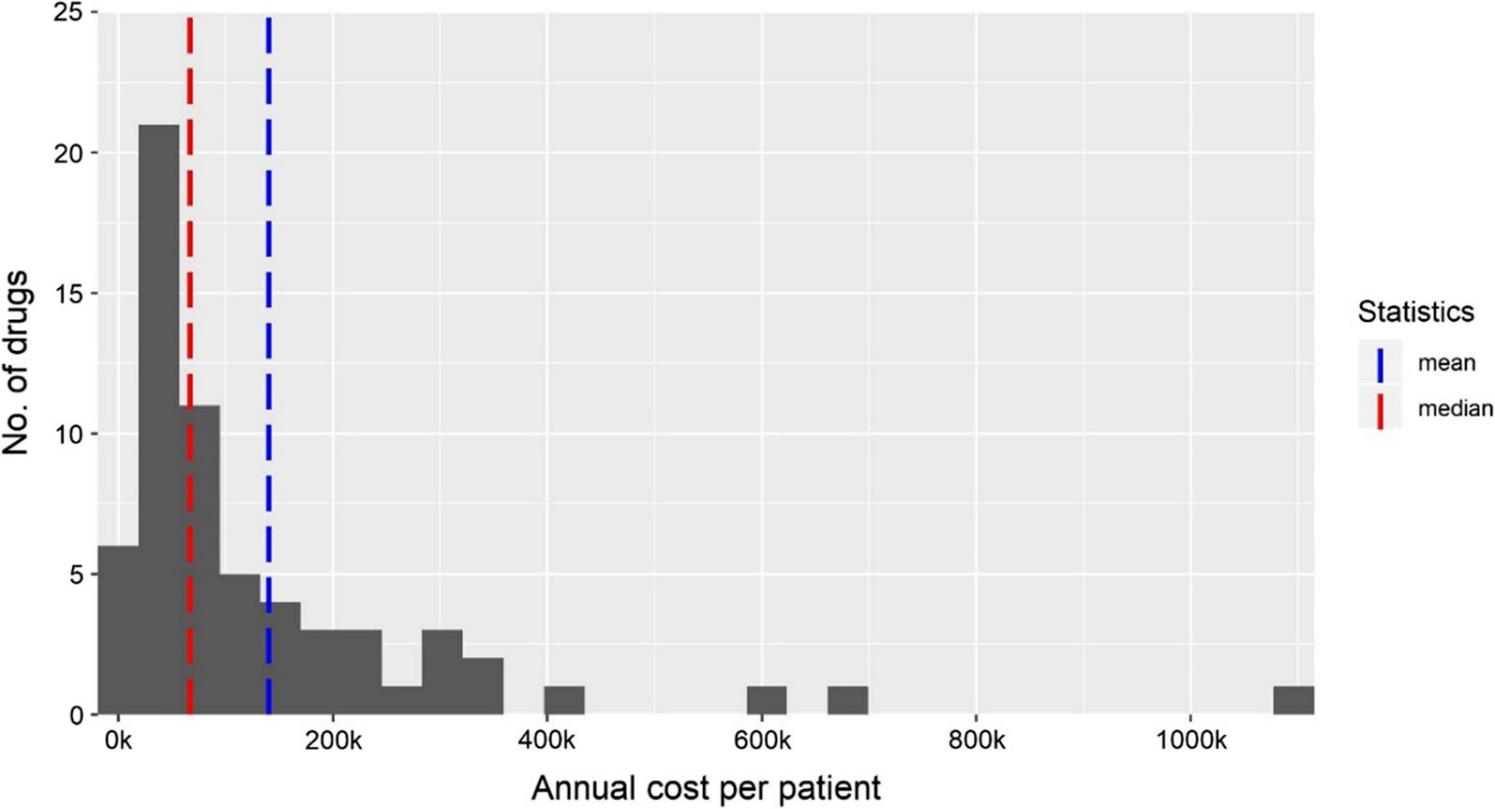
Distribution of annual treatment cost (thousands euros)

The number of orphan drugs that concluded the P&R process in Italy during the period under review is 69, with a maximum of 19 in 2017 and a minimum of 5 in 2014 (Table 1). Thirty-five of these belong to ATC L. Of these drugs, 58 obtained a favourable opinion for reimbursement and concluded the price negotiation, for 20 the P&R decision is still pending, 10 are not reimbursed and 1 was withdrawn. Table 1 also reports some descriptive statistics about the variables used in the analysis. The annual treatment cost ranges from 3.9 k euros to 1.1 million euros, as shown in Fig. 1.

**Table.1:** Orphan drugs approved in 2014–2019 per ATC, their reimbursement status and descriptive statistics about the variables used in the analysis

ATC	Total (89)	Reimbursed (58)	Not Reimbursed (11)	Ongoing negotiation/withdrawn (20)
A—alimentary tract and metabolism	16	9	2	5
B—blood and blood forming organs	7	2	2	3
C—cardiovascular system	4	2	0	2
D—dermatologicals	2	1	0	1
J—anti-infective agents for systemic use	7	5	1	1
L—antineoplastic and immunomodulating agents	38	31	4	3
M—musculoskeletal system	3	3	0	0
N—nervous system	3	1	1	1
R—respiratory system	3	1	1	1
S—sensory organs	5	3	0	2
H—systemic hormonal preparations (excl. sex hormones and insulins)	1	0	0	1

Year of P&R in Italy (GU)	2014	2015	2016	2017	2018	2019
TOT num of products *(negotiation concluded)*	5	8	14	19	13	10

Variable	Min	1st Qu	Median	Mean	3rd Qu	Max	NA’s
Annual treatment cost (€)	3934	44,959	66,601	140,606	165,465	1,100,066	0
Prevalence per 100 k population	0.09	1.30	5.31	10.89	12.75	114.10	0
RCT	0.00	0.00	1.00	0.65	1.00	1.00	3
ASMR	1.00	3.00	4.00	3.89	4.50	5.00	8

*ASMR* Amélioration du Service Médical Rendu, *GU* Gazzetta Ufficiale della Repubblica Italiana, *NA's* Not available, *Qu* Quartile, *RCT* Randomized Clinical Trial

Given the skewed distribution of the cost, we opted to run the main analysis with its logarithmic transformation. The mean prevalence of the target population is 10.89 per 100 k population, its quartiles going from 1.3 to 12.75. Thirty-nine out of 58 reimbursed drugs have an RCT as a pivotal study, while 3 approvals have solely bibliographic support for efficacy and safety. The others were approved with studies different from an RCT. As for the ASMR, the median value is 4 (minor added value), with only three drugs having values lower than 3 (i.e. major or important added value), and 8 drugs not having that information available.

**Fig. 2:**
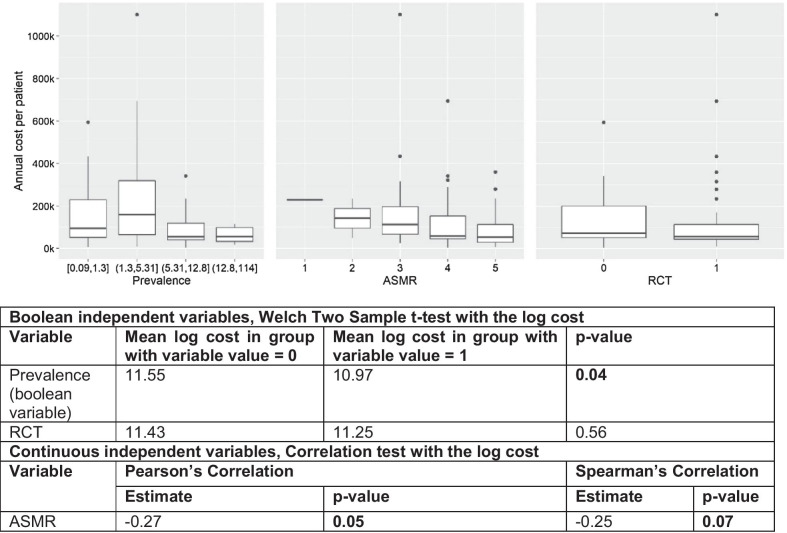
Correlation between the independent variables and the annual treatment cost (thousand euros). In bold, correlations significant at 10%. *ASMR* Amélioration du Service Médical Rendu, *RCT* Randomized Clinical Trial

Figure 2 shows the relation between the independent variables and the cost. The graph with the relation between the ASMR and the cost appears to support the hypothesis, i.e., greater values (lower added benefit) correspond to lower costs. We cannot state the same on the RCT-cost relationship: there are more outliers with higher costs in the case of RCTs, even though the box of cost values in case of no RCTs is wider. As for prevalence, by decomposing the variable in its quartiles we find a big difference in the cost before and after the median value. If we consider low levels of prevalence, we find an unexpected positive correlation between the cost and the prevalence. If we go through rare diseases with higher prevalence, the correlation is negative. For this reason, in the rest of the analysis we decided to treat the prevalence as a Boolean variable, with a value of 1 if greater than its median, 0 otherwise. The table under Fig. 2 reports findings from t-tests and correlation tests among independent variables and the logarithmic transformation of the cost. Using the Boolean variable for prevalence, the negative relation between the prevalence and the cost is confirmed: medicines with a prevalence below the median value have a higher annual treatment cost.

From the cluster analysis, we selected four clusters. Table 2 reports their characteristics in terms of the values assumed by our three variables. Based on this summary, it seems that the four clusters express all the combinations between prevalence and RCT, and for the ASMR, cluster 1 and cluster 3 present the lowest values (higher additional value). The annual treatment cost reveals that cluster 2 and cluster 4 are the ones with the lowest values, which is confirmed by the coefficients of a multivariate regression run with cluster being a categorical variable. From the cluster analysis, we cannot deduct with certainty which factor is playing the greatest role in determining the cost, but it appears that ASMR is the most important driver among the ones considered. In fact, the two clusters with higher costs (1 and 3) have opposite combinations of RCT and prevalence, while they both have the lowest values of ASMR. For this reason, we opted to run also univariate and multivariate regression analysis.

**Table.2:** Results of the cluster analysis

Cluster	Prevalence	Number of medicines	ASMR	RCT	Annual treatment cost (€)	Regression coefficient, 95% confidence interval
1	Low	13	Mean: 3.77	Yes	294,514	
2	Low	11	Mean: 3.91	No	107,297	− 1.06 (− 1.82, − 0.31)
3	High	6	Mean: 3.83	No	187,370	− 0.21 (− 1.12, 0.69)
4	High	22	Mean: 4.09	Yes	63,855	− 1.26 (− 1.9, − 0.62)

Cluster analysis based on Gower distance and Partition Around Medoids algorithm. Low/high prevalence = below/above median; no/yes RCT = no RCT/all RCT. 52 medicines were included (the ones for which we have complete information on RCT and ASMR). *ASMR* Amélioration du Service Médical Rendu, *RCT* Randomized Clinical Trial

Table 3 illustrates the results of the regressions. In the univariate analysis (regressions 1, 2, and 3), the prevalence and ASMR have, as expected, a negative relation with the annual treatment cost and these results are significant from a statistical viewpoint. The correlation with RCT is not significant. Regression 4 reports the multivariate model. Coefficients for prevalence and ASMR, all other things being equal, are confirmed even if the ASMR does not appear to be significant anymore. The same regression confirms a positive, but not significant from a statistical viewpoint, relation between RCT and costs, holding other variables constant.

**Table.3:** Results of the regression analyses

	Dependent variable: Log cost					
	(1)	(2)	(3)	(4)	(5)	(6)
Prevalence	− 0.582** (− 1.122, − 0.041)			− 0.526* (− 1.101, 0.049)	0.829* (− 0.073, 1.731)	1.197** (0.185, 2.210)
RCT		− 0.176 (− 0.739, 0.387)		0.141 (− 0.466, 0.748)	1.029*** (0.301, 1.758)	0.779* (− 0.002, 1.561)
ASMR			− 0.338** (− 0.665, − 0.011)	− 0.287 (− 0.625, 0.052)	− 0.225 (− 0.530, 0.080)	− 0.203 (− 0.533, 0.127)
ATC l						0.091 (− 0.685, 0.867)
Class H of eligibility						− 0.205 (− 0.955, 0.545)
2015						1.468** (0.341, 2.595)
2016						0.908 (− 0.187, 2.004)
2017						1.376*** (0.399, 2.354)
2018						1.110** (0.097, 2.124)
2019						0.888 (− 0.160, 1.937)
Prevalence * RCT					− 2.016*** (− 3.118, − 0.915)	− 2.187*** (− 3.338, − 1.037)
Constant	11.554*** (11.168, 11.939)	11.430*** (10.976, 11.884)	12.582*** (11.277, 13.886)	12.653*** (11.260, 14.047)	11.936*** (10.629, 13.244)	10.885*** (9.236, 12.534)
Observations	63	60	55	52	52	52
R^2^	0.068	0.006	0.072	0.123	0.312	0.449

*ASMR* Amélioration du Service Médical Rendu, *ATC* Anatomical Therapeutic Chemical (Classification), *Log cost* Logarithmic transformation of annual treatmente cost, *RCT* Randomized Clinical Trial

*p < 0.1; **p < 0.05; ***p < 0.01

Interesting insights can be gained from regression 5, where an interaction term between the prevalence and RCT was added.

**Fig. 3:**
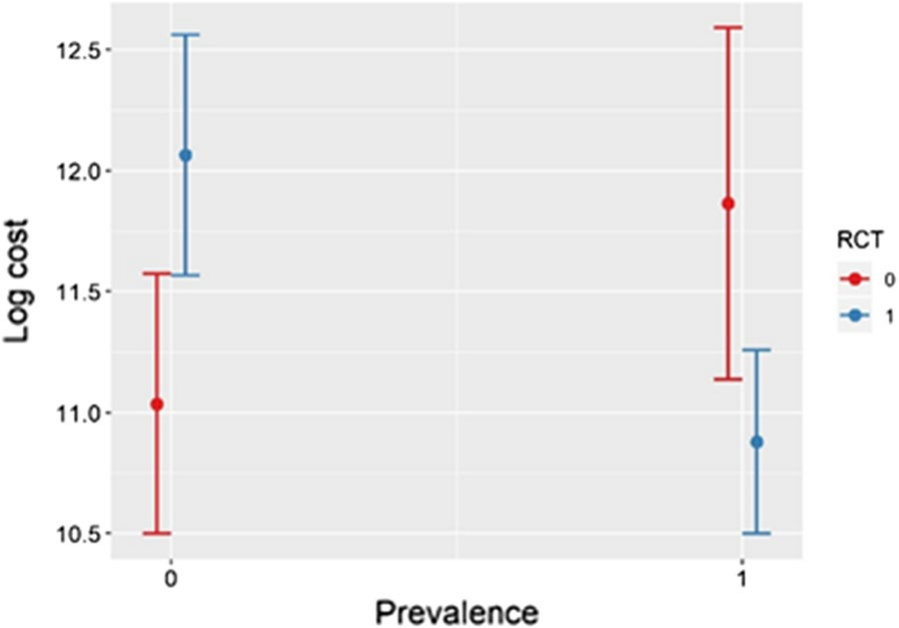
Costs, prevalence and RCT. Prevalence 0/1 = below/above the median value; RCT 0/1 = RCT No/Yes

Results show that when the prevalence is below the median value, costs are higher for RCT-driven orphan drugs (Fig. 3). The opposite occurs if the prevalence is above the median value. It thus seems that the RCTs may have a major impact within medicines targeting low prevalence disease: having an RCT as a pivotal study could play a more significant role in the case of drugs referring to small populations, with the prevalence Boolean variable having value 0. The inclusion of this interaction resulted in statistically significant results for RCT, and an important increase of R^2^.

Eventually, we inserted some control variables in regression 6 to control for potential confounding factors, such as the class of reimbursement, ATC level, and reimbursement year. It emerges that some reimbursement years have significant coefficients, while others do not. Even if they do not change the direction of the other coefficients, they do affect their significance and magnitude, as could be expected given the small sample size of the study.

## Discussion

The present paper investigated the role played by the dimension of the target population, the existence of an RCT, and the added therapeutic value on the price of orphan drugs in Italy.

Most of our findings seem to support the expected results. In general, the higher is the prevalence, the lower is the annual treatment cost, thus confirming a trade-off between prices and the target population, in alignment with previous contributions. It has to be stressed that the analysis relies on estimates of the target population included in the P&R dossier submitted by the relevant company. These estimates are based on the indication for which the company requires the reimbursement, but they do not necessarily coincide with the actual indication covered by the Italian National Health Service. Numbers for the latter, if eventually different from the former, may influence the P&R negotiation, but they are not necessarily available.

The added therapeutic value score, as measured by the French ASMR (with lower scores for higher added therapeutic value) is, as expected, negatively associated with the annual treatment cost, but its statistical significance disappears, moving from univariate and cluster analyses to multiple regression. It seems that the dimension of the target population (i.e. budget impact considerations) prevails on the added therapeutic value (which inspires a value-based pricing approach) as a price driver when both are investigated simultaneously.

The existence of an RCT, which was used as a proxy of the quality of the evidence, is never significant before introducing interaction with prevalence. The availability of an RCT pivotal study is positively correlated with the costs for medicines targeted to lower prevalence, but this correlation becomes non-significant when the prevalence is higher. This result is quite controversial. On the one hand, RCTs are more complex with low numbers. The choice of carrying out an RCT when the prevalence is low could be considered an important effort towards a higher quality of the evidence, that could be presumed to be correlated with the cost. On the other side, when the prevalence is too low (e.g. ultra-rare diseases) the presence of an RCT could be methodologically implausible and we may expect a negative impact on the perceived value and prices if prices are value-based.

Our results seem consistent with other findings. As it was mentioned before our analysis supports the trade-off between annual treatment cost and volumes and the correlation between annual treatment costs and added therapeutic value. Other variables (such as the class of reimbursement, ATC level) do not appear to have an impact on prices. It emerges that some reimbursement years have significant coefficients, while others do not. The correlation with reimbursement years could be associated with a tougher approach to cost-containment when this (negative) correlation was found. Findings of the above-mentioned French study [17] are quite similar, with only two important differences:

- variables other than volumes and added therapeutic value played a more important role: e.g. the ATC classification and some others not considered in our study, including the availability of other treatments and the delay between Health Technology Assessment (HTA) and commercialization, are correlated with orphan drugs prices;
- the French study did not investigate the interaction between explanatory variables, like the one we reported, i.e. RCT and prevalence. This highlighted the interesting, but controversial, finding that the presence of an RCT is associated with higher annual treatment costs when the target population is smaller.

Our study has three main limitations.

The first and most important one is the limited number of observations which drove our choice to consider a limited set of explanatory variables. For example, we used RCT as a proxy of quality. We also collected data on other variables that may detect the quality, but we decided to exclude them, either not to lose a degree of freedom (e.g. blinding) or because they are much more discretional (e.g. clinical validation of surrogate endpoints). Moreover, as it was mentioned before, other variables included in the above-mentioned French study, like disease severity [17], were not used because their measurement is difficult and the distinction between severe/not severe disease on the grounds of the Public summary of opinion on orphan designation is too simplistic. We are also aware that prices could be influenced by the level of unmet need other than the added therapeutic value, but its measurement is controversial [21] and in Italy, it is graded, like the added therapeutic value, only for medicines for which the innovativeness status is required.

The second limitation is that we relied on annual cost estimates, which may underestimate the cost of chronic treatments. The main problem of chronic treatments is that, in most circumstances, their mean duration is not available and we preferred to use annual treatment cost estimates, like other studies [17].

Finally, we relied on the added therapeutic value on the French grades for ASMR whose evaluation is done in comparison with products of the same pharmaco-therapeutic class that are already reimbursed in France. The rationale of this choice was discussed in the ‘Methods’ paragraph: we could not rely on the Italian appraisals on the added therapeutic value, since it is ranked and published only for medicines for which pharmaceutical companies have applied for innovativeness. Innovativeness was appraised for 38% of the medicines/indications included in our analysis, while 67% of appraised medicines got a full or conditional innovativeness status. The added therapeutic value was systematically ranked one grade better in Italy than in France, with most of the minor and moderate added value in France appraised as moderate and important in Italy respectively. As a consequence, the relative position among the candidate products was the same across France and Italy, with the Italian Medicines Agency providing better ranking than the French Transparency Committee regarding the evaluation of the grade of innovativeness.

## Conclusions

Despite its limitations, this paper provides some important insights into orphan drugs pricing and reimbursement in Italy.

Our results suggest that both value-based pricing and sustainability (dimension of the target population and budget impact) issues are considered: the former is supported by a positive association between the added therapeutic value and the annual treatment cost, the latter by a negative association of annual treatment costs with the dimension of the target population. However, the trade-off between prices and expected volumes appears to be supported by the statistical analyses, whereas the positive correlation between added value and annual treatment costs disappears when it is assessed within a multiple regression model. The role played by the quality of the evidence is much more controversial. It seems that those who assess, appraise and negotiate P&R, are aware of the difficulty of providing robust evidence for orphan drugs and less influenced by the quality of the evidence, although we could not rely on a counter-analysis for non-orphan medicines.

The literature found that in most European countries orphan medicines undergo the same HTA process of non-orphan drugs [22]: the current practice in Italy suggests that pricing for orphan drugs depends on the dimension of the target population and the added therapeutic value which are variables taken into account also for other medicines. Regardless of the debate on whether to opt for a specific model or simple customization of orphan drugs in the P&R framework, more transparent and reproducible assessment and P&R negotiation processes are needed in Italy. This paper has contributed to highlight the implicit drivers of this process.

## Data Availability

The datasets during and/or analysed during the current study are available from the corresponding author on reasonable request, with exception of the annual treatment cost that has been elaborated using confidential data (hidden discounts and managed entry agreement contracts).
